# The level and prevalence of depression and anxiety among patients with different subtypes of irritable bowel syndrome: a network meta-analysis

**DOI:** 10.1186/s12876-020-01593-5

**Published:** 2021-01-07

**Authors:** Zhichao Hu, Meixuan Li, Liang Yao, Yinshu Wang, Enkang Wang, Jianye Yuan, Fengyun Wang, Kehu Yang, Zhaoxiang Bian, Linda L. D. Zhong

**Affiliations:** 1 Hong Kong Chinese Medicine Clinical Study Center, School of Chinese Medicine, Hong Kong Baptist University, 7 Baptist University Road, Kowloon, 999077 Hong Kong SAR China; 2 Evidence-Based Medicine Center, School of Basic Medical Sciences, Lanzhou University, Lanzhou, 730000 China; 3 Department of Health Research Methods, Evidence and Impact, McMaster University, Hamilton, ON L8S 4L8 Canada; 4 Institute of Digestive Diseases, Longhua Hospital, Shanghai University of Traditional Chinese Medicine, Shanghai, 200032 China; 5 Xiyuan Hospital, China Academy of Chinese Medicinal Sciences, Beijing, 100091 China

**Keywords:** Irritable bowel syndrome, Depression, Anxiety, Meta-analysis

## Abstract

**Background:**

Irritable bowel syndrome (IBS) is a very common functional bowel disorder. However, the difference of depression and anxiety comorbidities among different IBS subtypes is still not well evaluated. This study aims to investigate the difference in the level and prevalence of depression and anxiety among healthy controls and patients with different subtypes of IBS.

**Methods:**

PubMed, EMBASE and the Cochrane library were searched systematically until August 17, 2020. Studies that investigated depression and/or anxiety levels or prevalence among different IBS-subtype patients measured at baseline or the same point were included. Network meta-analysis was conducted to analyze standardized mean difference (SMD) of anxiety and depression levels, and single arm meta-analysis was performed for prevalence of anxiety and depression among different IBS subtypes.

**Results:**

Eighteen studies involving 7095 participants were included. Network meta-analyses results showed healthy controls had a lower level of depression than IBS with mixed symptoms of constipation and diarrhea (IBS-M) [SMD =  − 1.57; 95% confidence interval (CI) − 2.21,  − 0.92], IBS with constipation (IBS-C) (SMD =  − 1.53; 95% CI − 2.13,  − 0.93) and IBS with diarrhea (IBS-D)(SMD =  − 1.41; 95% CI − 1.97,  − 0.85), while no significant difference was found between IBS unclassified (IBS-U) and healthy controls (SMD =  − 0.58; 95% CI  − 2.15, 1.00). There was also no significant difference in the level of depression among different IBS subtypes patients. The results of anxiety were similar to depression. Ranking probability showed that IBS-M was associated with the highest level of depression and anxiety symptoms, followed by IBS-C/IBS-D and IBS-U. Single-arm meta-analysis showed IBS-C had the highest prevalence of depression (38%) and anxiety (40%), followed by IBS-D, IBS-M and IBS-U.

**Conclusion:**

The results indicated that IBS-M was more likely to be associated with a higher level of depression and anxiety, and the prevalence of depression and anxiety in IBS-C was highest. The psychological screening and appropriate psychotherapy are needed for patients with IBS-C, IBS-D and IBS-M instead of IBS-U.

## Background

Irritable bowel syndrome (IBS) is a prevalent, costly and potentially disabling functional bowel disorder characterized by recurrent abdominal pain or changes in bowel habits [1]. The global prevalence of IBS is estimated to be 11.2%, but regionally it varies between 1.1 and 45% of the general population [2], and IBS can impose a major cost burden on the healthcare services and society [3, 4]. The estimating annual cost per patient in U.S. is $742–$7547, and the annual cost is around $1.7 billion to $10 billion in direct medical costs [3, 5]. On the basis of the Rome IV criteria, IBS is classified into four subtypes IBS with diarrhea (IBS-D), IBS with constipation (IBS-C), IBS with mixed symptoms of constipation and diarrhea (IBS-M), or IBS unclassified (IBS-U) according to patients’ reports of the proportion of time they have hard or lumpy stools versus loose or watery stools [6].

IBS patients often suffer from a high burden of depression and anxiety [7]. According to a clinic-based study, the prevalence of depression and anxiety in irritable bowel syndrome patients is 37.1 and 31.4% respectively [8]. However, the pathophysiology of IBS is still unclear. “Biopsychosocial Conceptual Model” and “Multi-Dimensional Clinical Profile” are emphasized according to the Rome IV criteria, showing that psychosocial factors and physiology states influence the presentation of functional gastrointestinal disorders (FGIDs). As for the biopsychosocial aspects, complex factors such as environmental, psychological and biological factors interactively play an important role in the development and maintenance of FGIDs [9, 10].

Some meta-analyses investigated the relationship between IBS and mental disorders such as depression and anxiety previously [7, 11–13]. A pairwise meta-analysis including 10 studies evaluated the mood changes between healthy controls and IBS patients. It indicated that the depression and anxiety levels were higher in IBS patients than in healthy controls (SMD = 0.80; 95% CI 0.42–1.19 and SMD = 0.76; 95% CI 0.47–0.69 respectively) [7]. Similar results were revealed in another three studies about depression or anxiety on the symptoms or prevalence of IBS patients [11–13]. Although previous reviews found that patients with IBS had a higher risk of depression or anxiety compared with healthy controls under directly comparing, the difference of depression and anxiety among different IBS subtypes was still not well evaluated.

Understanding the comorbidity between IBS and mental disorders will be used to explore the complex pathophysiology of IBS, and further prevent trigger symptom flares so as to develop an individualized treatment plan among patients with different IBS subtypes. There was a need for a network meta-analysis ranking the level of depression and anxiety of different IBS subtypes. Therefore, this study aimed to: (1) compare the level of depression or anxiety among healthy controls and different IBS subtypes patients; (2) define which subtype of IBS is more likely associated with depression or anxiety; (3) Investigate the prevalence of depression and anxiety in different IBS subtypes patients.

## Methods

This review was reported according to the Preferred Reporting Items for Systematic Review and Meta-Analyses Statement [14]. This study was registered with PROSPERO, Number CRD42019124174.

### Search strategy

A systematic search was conducted in PubMed, EMBASE and Cochrane library (the sixth edition, 2018) by two independent reviewers (H.Z.C. and L.M.X.) from their inception to June 2018, and the last search data was updated on Aug 17, 2020. The following key terms were used in the search strategies: (irritable bowel syndrome OR IBS) AND (depression OR depressive disorders OR dysthymic disorder OR anxiety OR anxiety disorders OR anxiousness OR mood disorders). The search strategies were well designed and varied from different databases. The details of search strategies were presented in Appendix 1.

### Selection criteria

Studies that met the following criteria were included: (1) studies that investigated anxiety and/or depression level in different IBS subtypes patients measured at baseline or the same time point without design restrictions; (2) studies that assessed anxiety and depression through validated psychometric measures such as Hospital Anxiety and Depression Scale (HADS), Beck's Depression Inventory (BDI), Self-Rating Depression Scale (SDS) and others; (3) patients met established diagnostic criteria of IBS, including ROME I, II, III, IV.

The patients under the diagnoses of IBS due to the general medical condition, post‐infectious IBS or related serious comorbidities were excluded.

### Selection of studies and data extraction

A pre-made form with characteristics (first author, publication year, country, study design, sample size, age, gender, IBS diagnostic criteria, anxiety and depression scale, etc.) and target outcomes (anxiety level, depression level, depression prevalence, anxiety prevalence, etc.) were prepared. Two reviewers (H.Z.C. and L.M.X.) independently screened the abstracts and full-texts and extracted data. Rome IV criteria used the term “IBS with mixed bowel habits (IBS-M)” instead of “IBS alternating type (IBS-A)”, so we combined the data of these two types. The third reviewer (Z.L.D.) was invited for rechecking data extraction, and any disagreements were discussed among three reviewers.

### Assessment of methodological quality

Two authors (Y.L. and L.M.X.) independently assessed the quality of included cohort studies or case–control studies according to the criteria developed by the Newcastle–Ottawa Scale (NOS) [15]. The NOS is widely used for cohort study and case–control study quality assessment focusing on the selection, comparability, exposure or outcome. For cross-sectional studies, the quality of cross-sectional study was assessed using criteria recommended by the Agency for Healthcare Research and Quality (AHRQ) [16]. The criteria were divided into 11 items such as source of information, inclusion/exclusion criteria, time period for identity, subjects consecutive, evaluators masked, quality assurance assessments and so on. For each question, a score of 1 for “Yes” or 0 for “No”/ “Unclear” was given, out of a total score of 11. And studies achieving 6 or more points were considered to be of high quality. Any disagreements were resolved via discussion among the author group.

### Statistical analyses

A frequentist model was used in this network meta-analysis to combine direct and indirect evidence from all included studies. Stata 13.0 software (Stata Corporation, College Station, Texas, USA) was used to complete all analyses. First, standard pairwise meta-analysis was conducted with a random-effects model. Second, a network meta-analysis was processed using the mvmeta package of the Stata software, which was based on a multiple regression model. Evidence of inconsistency was checked using the node-splitting method. Random-effects pairwise and network meta-analyses were applied to obtain estimates for outcomes, and these estimates were presented as standardized mean differences (SMD) (continuous outcomes) with 95% confidence intervals (CIs), and a *P *value < 0.05 was considered statistically significant.

For all outcomes, network diagrams were used to summarize the evidence. The characteristics of included studies were summarized in a table and presented the comparisons in different tables. For outcomes, we also displayed the ranking probabilities of interventions by the surface under the cumulative ranking curve (SUCRA) which would show the best rank mostly approaching 1 [17]. For the included studies reporting the prevalence of depression and anxiety in different IBS subtypes patients, R 4.0.2 software was used to do a single arm Meta-analysis. This analysis took study effects into account, and the results were calculated by a binary random-effect method (Dersimonian-Laird). Forest plots were used to illustrate the prevalence with 95% CIs.

## Results

### Literature search

**Fig. 1:**
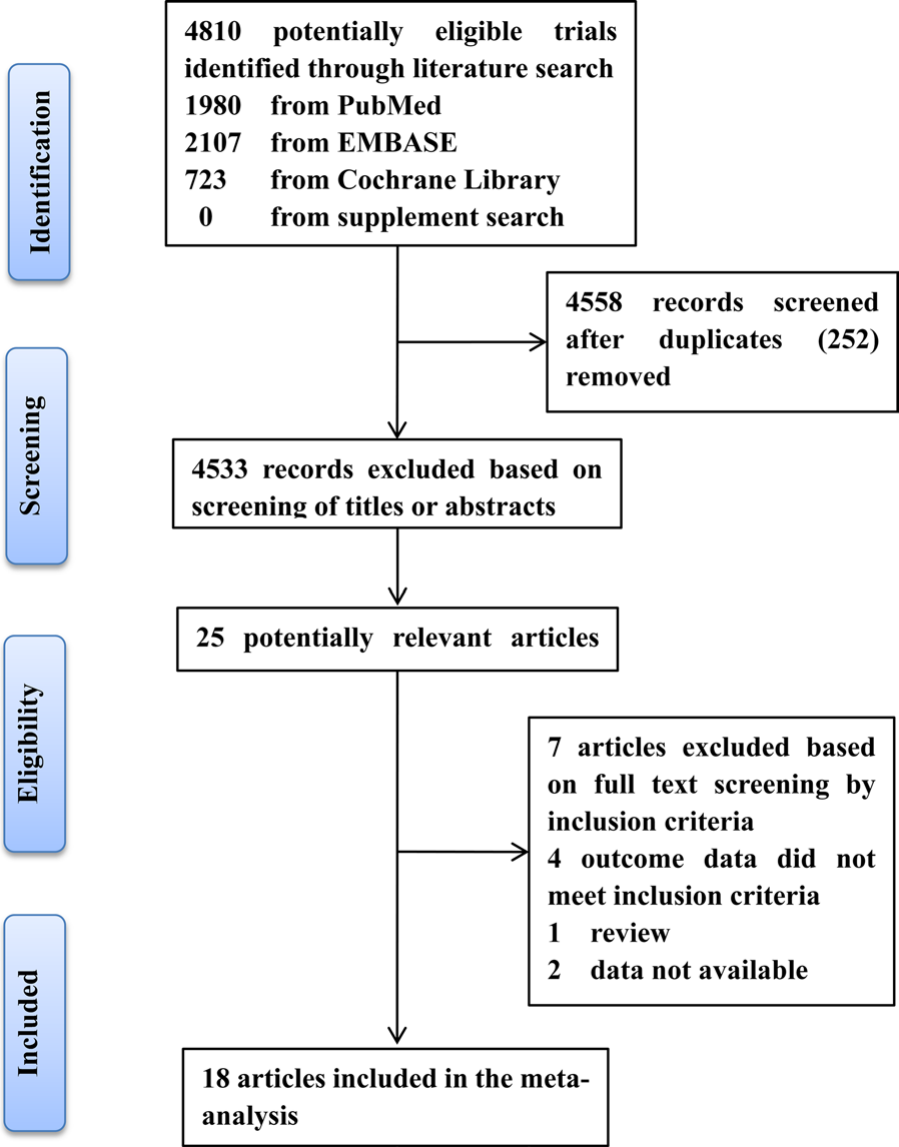
Flow diagram of the study selection process

The literature search yielded 4810 reports, of which 252 were excluded for duplication, and 4533 were excluded on the basis of titles or abstracts that were irrelevant to the topic, and seven were excluded from the remaining 25 literatures after reading the full texts. Finally, 18 studies [18–35] with 7095 participants were included for analysis, of which four studies were cohort studies, eight studies were case–control studies, the remaining six studies were cross-sectional studies. The PRISMA flow chart of literature studies for meta-analysis was illustrated in Fig. 1.

### Study characteristics

**Fig. 2:**
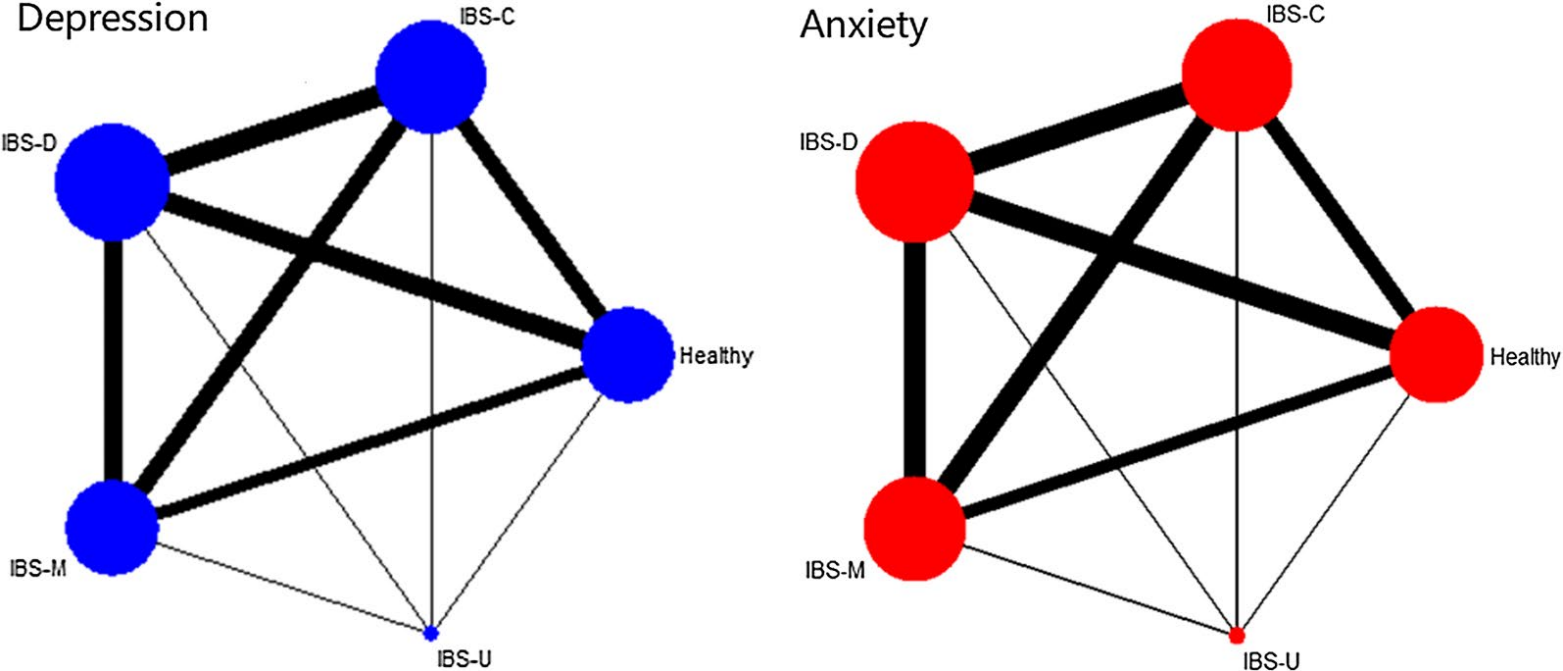
Network maps for depression and anxiety level

The baseline and general characteristics of the included studies were extracted and listed in Table 1. Eighteen studies were all published in English between 1998 and 2020, and the study samples ranged from 29 to 1987 participants. The level of depression and anxiety were compared in five groups (Healthy controls, IBS-D, IBS-C, IBS-M and IBS-U). Networks of eligible comparisons for the level of depression and anxiety were presented in Fig. 2. Twelve studies [18–21, 24, 26–28, 33, 34] used the HADS at baseline to measure the levels of anxiety and depression. IBS was diagnosed using the Rome III criteria in seven studies [18, 24–26, 29, 30, 34], Rome II criteria in nine studies [19–22, 27, 28, 31, 32, 35], one [33] used Rome IV criteria, one study [23] just mentioned they used Rome criteria. More details of included studies and the quality assessment results were showed in Table 1.

**Table 1:** Characteristics of included studies

Study	Country	Study design	Sample size	Age (years)	Gender (female, %)	IBS diagnostic criteria	Anxiety and depression scale	Quality
Camilleri et al. [28]	US	Cohort	Healthy (n = 41); IBS-C (n = 49); IBS-D (n = 44); IBS-M (n = 29)	Healthy:33.6 ± 1.6; IBS-C:38.6 ± 1.5; IBS-D:35.4 ± 1.6; IBS-M:37.2 ± 2.2	Healthy:100; IBS-C:100; IBS-D:93.2; IBS-M:100	Rome II	HADS	7
Cho et al. [27]	Korea	Case–control	Healthy (n = 91); IBS-C (n = 30); IBS-D (n = 63); IBS-M (n = 31)	Healthy:45.8 ± 15.2; IBS:43.3 ± 14.3	Healthy:46.2; IBS:49.2	Rome II	HADS	7
Jamali et al. [25]	Iran	Cross sectional	IBS-C (n = 75); IBS-D (n = 55); IBS-M (n = 120)	IBS-C:32.90 ± 10.1; IBS-D:29.12 ± 10.4; IBS-M: 31.97 ± 13.5	IBS-C:62.1; IBS-D:48.8; IBS-M:50	Rome III	BDI	7
Jones et al. [24]	Australia	Case–control	Healthy (n = 76); IBS-C (n = 60); IBS-D (n = 57); IBS-M (n = 51)	Healthy: 38.8 ± 12.4; IBS-C: 38.8 ± 12.6; IBS-D: 41.1 ± 13.6; IBS-M: 37.5 ± 13.3	Healthy:79; IBS-C:86; IBS-D:65; IBS-M:85	Rome III	HADS	7
Lee et al. [23]	China	Case–control	Healthy (n = 20); IBS-C (n = 20); IBS-D (n = 20)	Healthy:50.7 ± 17.4; IBS-C: 48.9 ± 16.3; IBS-D: 49.2 ± 15.6;	Healthy:30; IBS-C:30; IBS-D:30	NA	MMPI	6
Medeiros et al. [22]	Brazil	Case–control	Healthy (n = 8); IBS-C (n = 7); IBS-D (n = 6); IBS-M (n = 8)	Healthy:32.3(24–44); IBS:39.9(20–60)	Healthy:25.0; IBS:76.2	Roma II	HADRS	7
Miller et al. [21]	UK	Cohort	IBS-C (n = 296); IBS-D (n = 256); IBS-M (n = 448)	IBS:51.6(17–91)	IBS:80	Rome II	HADS	7
Seminowicz et al. [20]	Canada	Case–control	Healthy (n = 48); IBS-C (n = 15); IBS-D (n = 17); IBS-M (n = 19)	Healthy: 31.1 ± 12.3; IBS-C: 35.0 ± 13.4; IBS-D: 32.0 ± 5.46; IBS-M: 31.2 ± 10.9	NA	Rome II	HADS	8
Sugaya et al. [19]	Japan	Cross sectional	Healthy (n = 1881); IBS-C (n = 45); IBS-D (n = 61)	Healthy:19.77 ± 1.73; IBS:19.53 ± 1.87	Healthy: 52.2; IBS:56.3	Rome II	HADS	7
Thijssen et al. [18]	Netherlands	Cohort	Healthy (n = 137); IBS-C (n = 33); IBS-D (n = 52); IBS-M (n = 60); IBS-U (n = 9)	Healthy: 44.2 ± 19.3; IBS: 44.5 ± 16.3	Healthy: 61; IBS: 70	Rome III	HADS	8
De-Rong et al. [26]	China	Case–control	Healthy (n = 20); IBS-D (n = 42)	Healthy:28.9 (20–38); IBS-D:29.4 (22–40)	Healthy: 40; IBS-D: 35.7	Rome III	HADS	8
Mujagic et al. [34]	Netherlands	Case–control	Healthy (n = 94); IBS-C (n = 21); IBS-D (n = 34); IBS-M (n = 30); IBS-U (n = 6)	IBS:44.4 ± 1.6; Healthy:45.0 ± 2.0	IBS: 63.7; Healthy:58.5	Rome III	HADS	7
Ladep et al. [35]	Nigeria	Cross sectional	IBS-C (n = 59); IBS-D (n = 58); IBS-A (n = 15)	32.0 ± 9.4	56.8	Rome II	DSM-IV	7
Qin et al. [33]	China	Case–control	Healthy (n = 18); IBS-D (n = 40)	IBS-D:44.50 ± 9.27; Healthy:42.33 ± 12.81	IBS-D:25; Healthy:27.8%	Rome IV	HADS	8
Bruno et al. [30]	Italy	Cohort	IBS-C (n = 34); IBS-D (n = 37); IBS-M (n = 40)	IBS-C:47.6 ± 10.1; IBS-D:46.7 ± 11.7; IBS-M:45.6 ± 13.9	56.76	Rome III	HRSD/HRSA	7
Ford et al. [29]	Canada	Cross sectional	IBS-C (n = 175); IBS-D (n = 380); IBS-M (n = 509)	IBS-C:43.0 ± 16.1; IBS-D:41.8 ± 15.0; IBS-M:38.2 ± 14.7	IBS-C:80.6; IBS-D:69.5; IBS-M:77.0	Rome III	HADS	8
Mearin et al. [31]	Spain	Cohort	IBS-C (n = 160); IBS-D (n = 182); IBS-A (n = 175)	NA	IBS-C:83.1; IBS-D:66.3; IBS-A:79.4	Rome II	EuroQoL-5D	7
Okami et al. [32]	Japan	Cross sectional	IBS-C (n = 260); IBS-D (n = 116); IBS-A (n = 252)	NA	IBS-C:20.4; IBS-D:6.6; IBS-A:14.5	Rome II	HADS	7

NA, not applicable; HADS, Hospital Anxiety and Depression scale; MMPI, scales of the Minnesota Multiphasic Personality Inventory; HADRS, Hamilton Anxiety and Depression Rating Scale; SCL, symptom checklist; DSM, Diagnostic and Statistical Manual of Mental Disorders; BDI, Beck Depression Inventory

### The level of depression and anxiety in different IBS subtypes

#### Depression

**Fig. 3:**
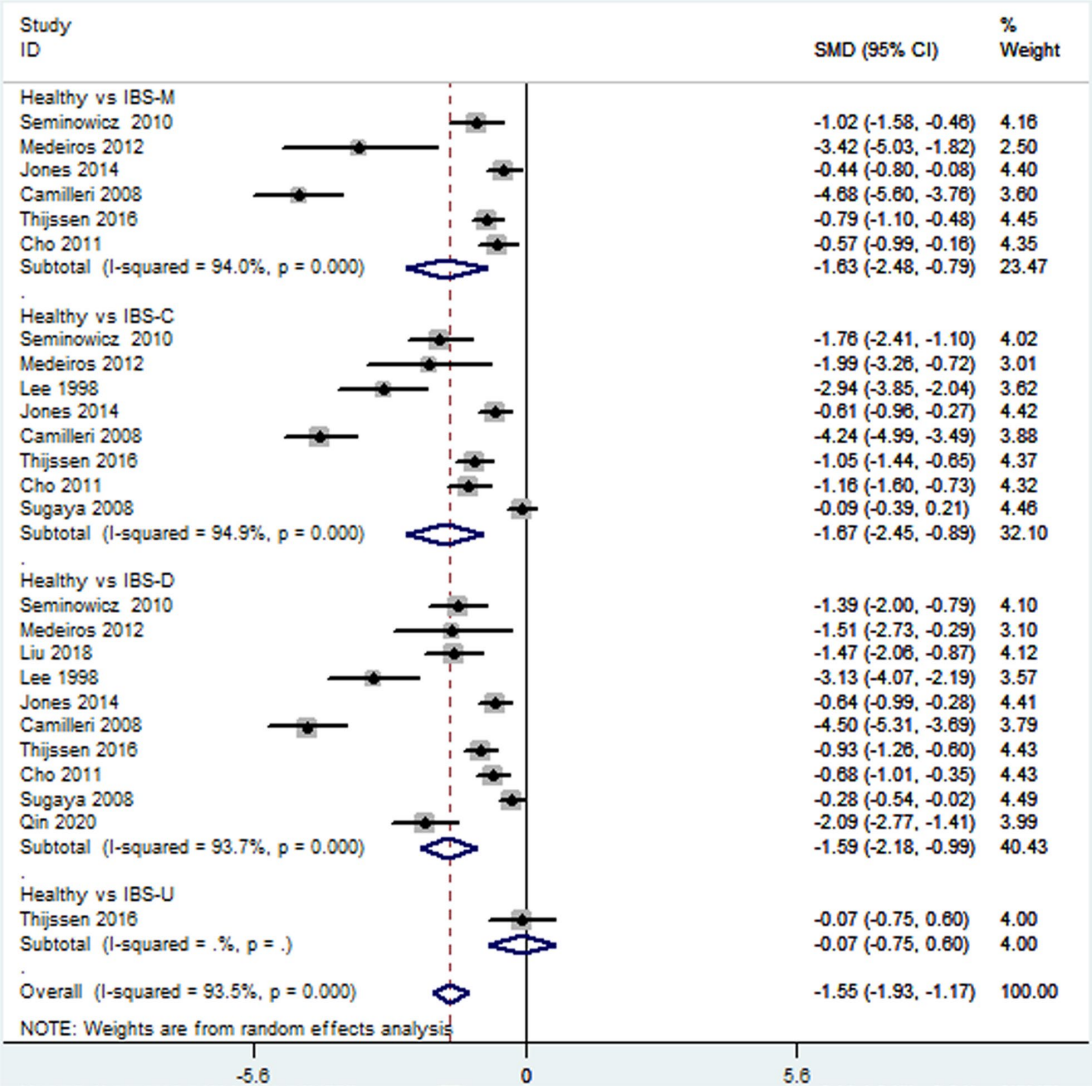
Direct comparison of depression level among different IBS subtypes and healthy controls

**Fig. 4:**
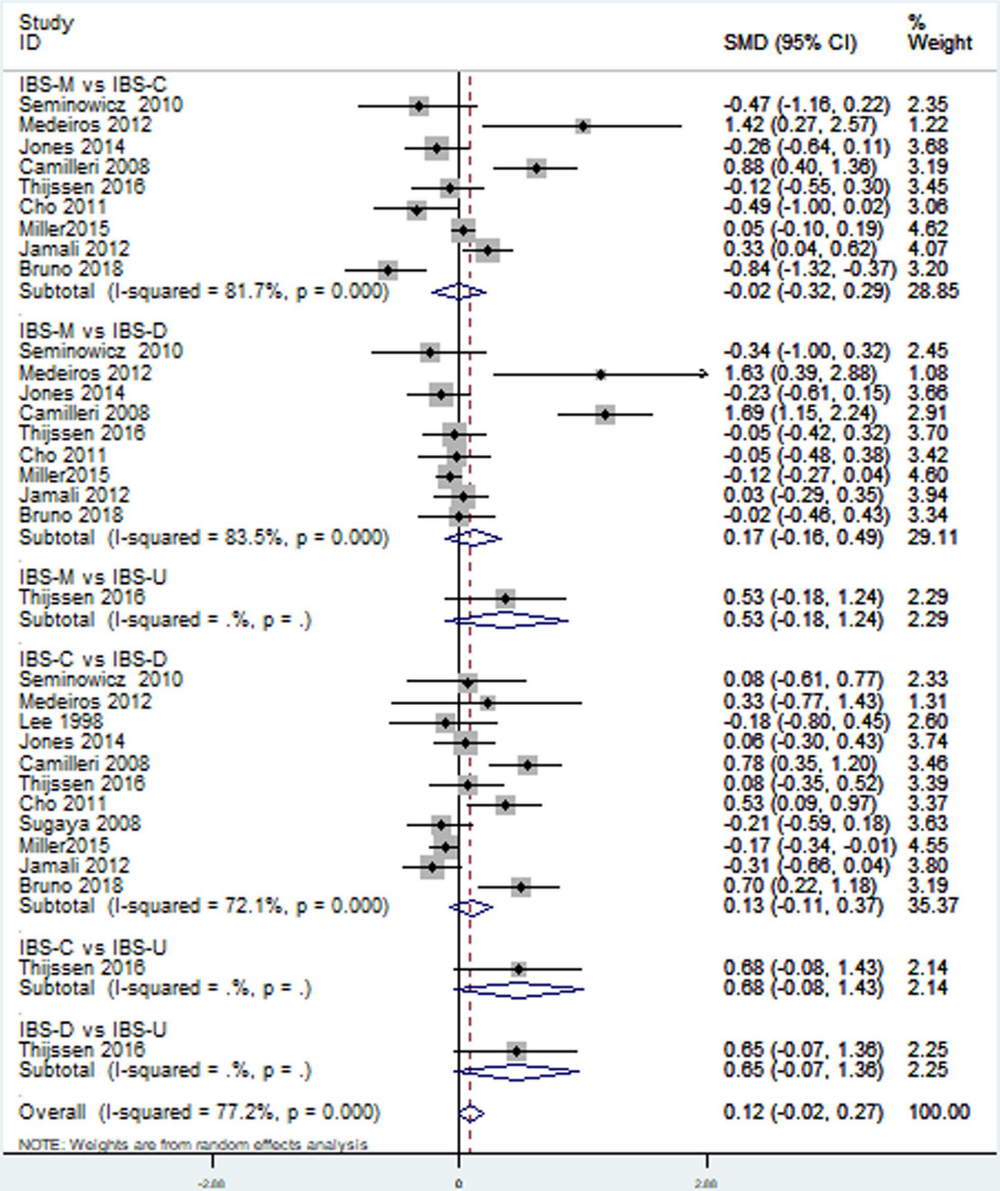
Direct comparison of depression level among different IBS subtypes

Thirteen [18–28, 30, 33] included studies reported depression level in patients with different IBS subtypes. Direct pairwise random-effects meta-analyses showed healthy controls had a lower level depression than IBS-M (SMD = ** − **1.63; 95% CI − 2.48,  − 0.79; *P* < 0.05), IBS-C (SMD = ** − **1.67; 95% CI − 2.45,  − 0.89; *P* < 0.05) and IBS-D (SMD = ** − **1.59; 95% CI − 2.18,  − 0.99; *P* < 0.05), while no significant difference was found between IBS-U and healthy controls (SMD = ** − **0.07; 95% CI − 0.75, 0.60; *P* > 0.05). There was also no significant difference among different IBS subtypes, IBS-M vs IBS-C (SMD = 0.02; 95% CI − 032, 0.29; *P* > 0.05, I^2^ = 81.7%), IBS-M vs IBS-D (SMD = 0.17; 95% CI − 0.16, 0.49; *P* > 0.05), IBS-M vs IBS-U (SMD = 0.53; 95% CI − 0.18, 1.24; *P* > 0.05), IBS-C vs IBS-D (SMD = 0.13; 95% CI − 0.11, 0.37; *P* > 0.05), IBS-C vs IBS-U (SMD = 0.68; 95% CI − 0.08, 1.43; *P* > 0.05), IBS-D vs IBS-U (SMD = 0.65; 95% CI − 0.07, 1.36; *P* > 0.05). More details were showed on Table 2, Figs. 3 and 4.

**Table 2:** Direct pairwise random-effects meta-analyses of outcomes

Interventions	Depression			Anxiety		
	SMD	95% CI	*P* value	SMD	95% CI	*P* value
Healthy vs IBS-M	− 1.63	− 2.48, − 0.79	0	− 1.8	− 2.70, − 0.90	0
Healthy vs IBS-C	− 1.67	− 2.45, − 0.89	0	− 1.34	− 2.02, − 0.65	0
Healthy vs IBS-D	− 1.59	− 2.18, − 0.99	0	− 1.36	− 1.95, − 0.78	0
Healthy vs IBS-U	− 0.07	− 0.75, 0.60	0.83	− 0.7	− 1.38, − 0.02	0.04
IBS-M vs IBS-C	− 0.02	− 0.32, 0.29	0.58	0.02	− 0.27, 0.30	0.42
IBS-M vs IBS-D	0.17	− 0.16, 0.49	0.28	− 0.00	− 0.11, 0.11	0.75
IBS-M vs IBS-U	0.53	− 0.18, 1.24	0.14	0.35	− 0.36, 1.05	0.34
IBS-C vs IBS-D	0.13	− 0.11, 0.37	0.55	0.03	− 0.18, 0.24	0.89
IBS-C vs IBS-U	0.68	− 0.08, 1.43	0.08	0.34	− 0.40, 1.08	0.37
IBS-D vs IBS-U	0.65	− 0.07, 1.36	0.08	0.34	− 0.37, 1.05	0.35

The results of the network analysis also showed healthy controls had a lower level depression than IBS-M (SMD = ** − **1.57; 95% CI − 2.21, ** − **0.92; *P* < 0.05), IBS-C (SMD = ** − **1.53; 95% CI − 2.13, ** − **0.93; *P* < 0.05) and IBS-D (SMD = ** − **1.41; 95% CI − 1.97, ** − **0.85; *P* < 0.05), while no significant difference was found between IBS-U and healthy controls (SMD = ** − **0.58; 95% CI − 2.15, 1.00; *P* > 0.05). There was also no significant difference among different IBS subtypes, IBS-M vs IBS-C (SMD = 0.04; 95% CI − 0.55, 0.62; *P* > 0.05), IBS-M vs IBS-D (SMD = 0.16; 95% CI − 0.42, 0.74; *P* > 0.05), IBS-U vs IBS-M (SMD = ** − **0.99; 95% CI − 2.56, 0.59; *P* > 0.05), IBS-D vs IBS-C (SMD = ** − **0.12; 95% CI − 0.66, 0.42; *P* > 0.05), IBS-U vs IBS-C (SMD = ** − **0.95; 95% CI − 2.52, 0.62; *P* > 0.05), IBS-U vs IBS-D (SMD = ** − **0.83; 95% CI − 2.39, 0.73; *P* > 0.05). More details were showed on Table 3.

**Table 3:** Network meta-analysis of the outcomes

Depression				
*Healthy*				
− 0.58 (− 2.15, 1.00)	*IBS-U*			
− 1.57 (− 2.21, − 0.92)	− 0.99 (− 2.56, 0.59)	*IBS-M*		
− 1.41 (− 1.97, − 0.85)	− 0.83 (− 2.39, 0.73)	0.16 (− 0.42, 0.74)	*IBS-D*	
− 1.53 (− 2.13, − 0.93)	− 0.95 (− 2.52, 0.62)	0.04 (− 0.55, 0.62)	− 0.12 (− 0.66, 0.42)	*IBS-C*

Anxiety				
*Healthy*				
− 0.89 (− 2.42, 0.64)	*IBS-U*			
− 1.43 (− 2.06, − 0.79)	− 0.54 (− 2.07, 0.99)	*IBS-M*		
− 1.35 (− 1.92, − 0.78)	− 0.46 (− 1.98, 1.06)	0.08 (− 0.49, 0.64)	*IBS-D*	
− 1.36 (− 1.97, − 0.75)	− 0.47 (− 1.99, 1.06)	0.07 (− 0.51, 0.65)	− 0.01 (− 0.55, 0.54)	*IBS-C*

#### Anxiety

**Fig. 5:**
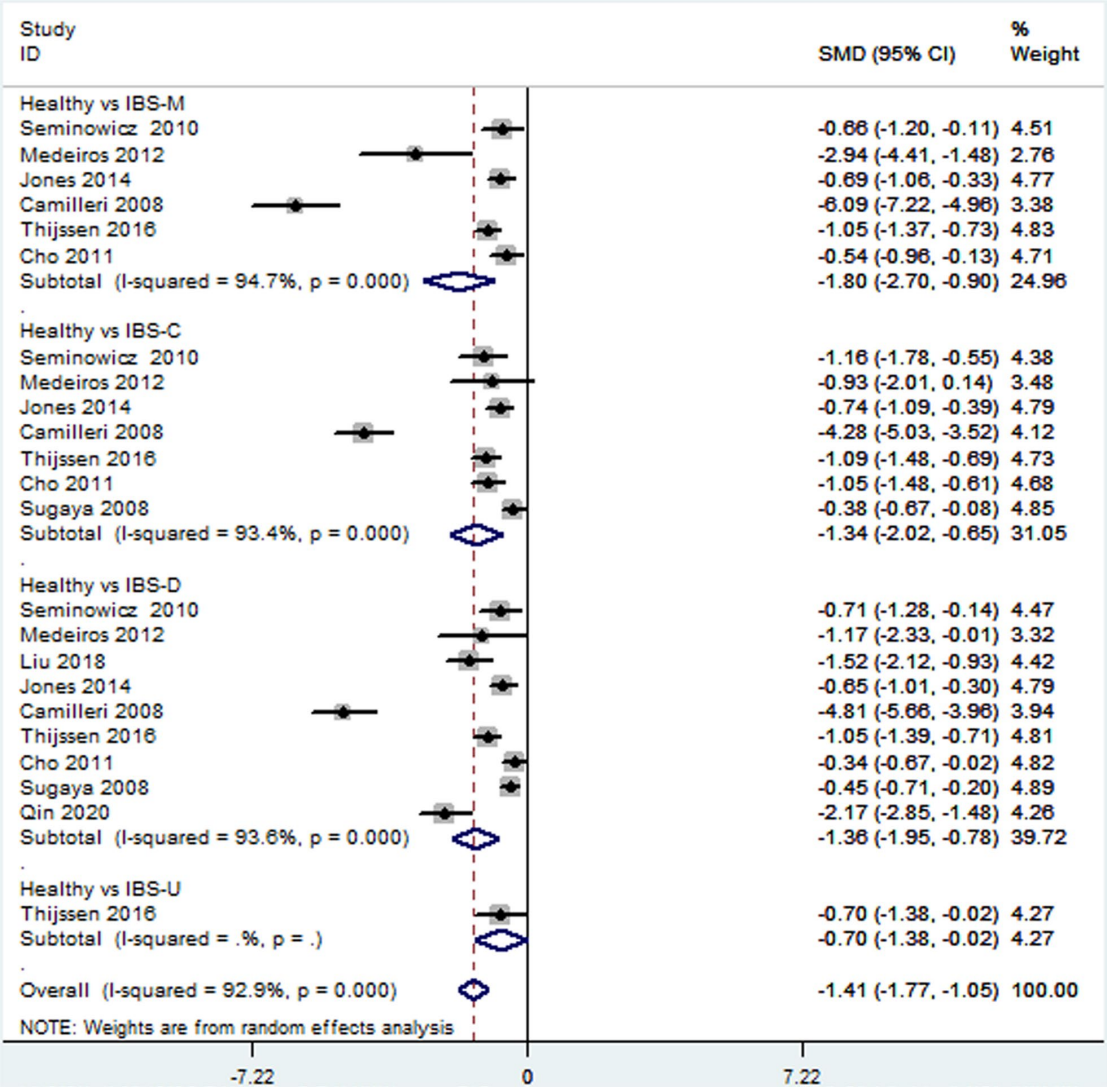
Direct comparison of anxiety level among different IBS subtypes and healthy controls

**Fig. 6:**
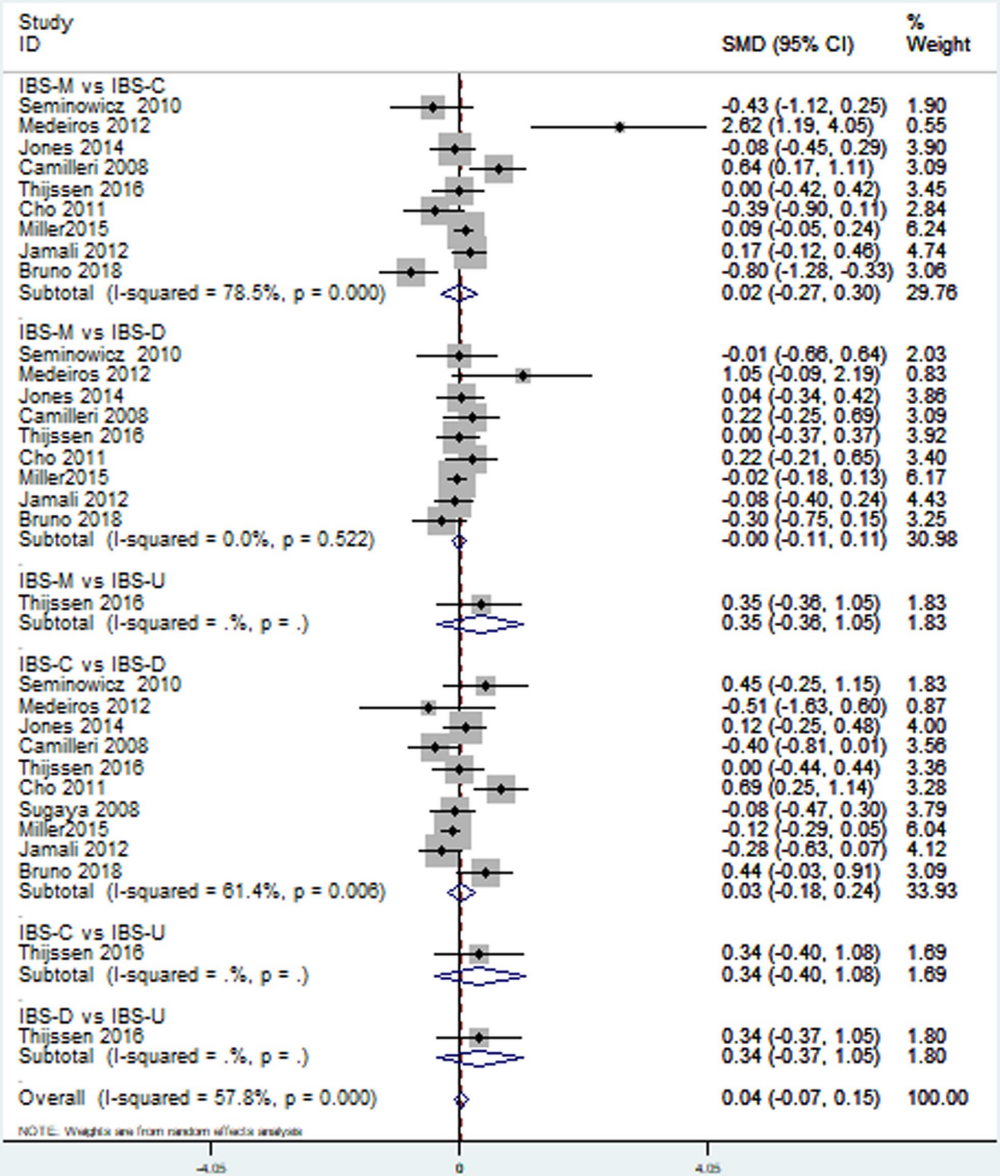
Direct comparison of anxiety level among different IBS subtypes

Twelve [18–22, 24–28, 30, 33] studies provided the data of the anxiety level in patients with different IBS subtypes. Direct pairwise random-effects meta-analyses showed that healthy controls had a lower level anxiety than IBS-M (SMD = ** − **1.80; 95% CI − 2.70, ** − **0.90; *P* < 0.05), IBS-C (SMD = ** − **1.34; 95% CI − 2.02, ** − **0.65; *P* < 0.05), IBS-D (SMD = ** − **1.36; 95% CI − 1.95, ** − **0.78; *P* < 0.05) and IBS-U (SMD = ** − **0.70; 95% CI − 1.38, ** − **0.02; *P* < 0.05). There was also no significant difference among different IBS subtypes, IBS-M vs IBS-C (SMD = 0.02; 95% CI − 0.27, 0.30; *P* > 0.05), IBS-M vs IBS-D (SMD = 0.00; 95% CI − 0.11, 0.11; *P* > 0.05), IBS-M vs IBS-U (SMD = 0.35; 95% CI − 0.36, 1.05; *P* > 0.05), IBS-C vs IBS-D (SMD = 0.03; 95% CI − 0.18, 0.24; *P* > 0.05), IBS-C vs IBS-U (SMD = 0.34; 95% CI − 0.40, 1.08; *P* > 0.05), IBS-D vs IBS-U (SMD = 0.34; 95% CI − 0.37, 1.05; *P* > 0.05). More details were showed on Table 2, Figs. 5 and 6.

The results of the network analysis also showed healthy controls had a lower level anxiety than IBS-M (SMD = ** − **1.43; 95% CI − 2.06, ** − **0.79; *P* < 0.05), IBS-C (SMD = ** − **136; 95% CI − 1.97, ** − **0.75); *P* < 0.05) and IBS-D (SMD = ** − **1.35; 95% CI − 1.92, ** − **0.78; *P* < 0.05), no significant difference was found between IBS-U and healthy controls (SMD = ** − **0.89; 95% CI − 2.42, 0.64; *P* > 0.05). There was also no significant difference among different IBS subtypes, IBS-M vs IBS-C (SMD = 0.07; 95% CI − 0.51, 0.65; *P* > 0.05), IBS-M vs IBS-D (SMD = 0.08; 95% CI − 0.49, 0.64; *P* > 0.05), IBS-U vs IBS-M (SMD = ** − **0.54; 95% CI − 2.07, 0.99; *P* > 0.05), IBS-D vs IBS-C (SMD = ** − **0.01; 95% CI − 0.55, 0.54; *P* > 0.05), IBS-U vs IBS-C (SMD = ** − **0.47; 95% CI − 1.99, 1.06; *P* > 0.05), IBS-U vs IBS-D (SMD = ** − **0.46; 95% CI − 1.98, 1.06; *P* > 0.05). More details were showed on Table 3.

### Inconsistency analyses

Node-splitting analysis showed that there were no inconsistencies between direct and indirect comparisons (*P* > 0.05).

### Rank probability

**Fig. 7:**
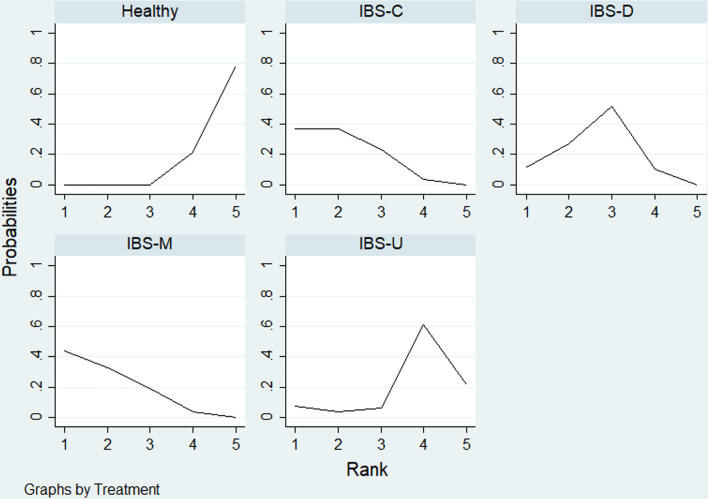
Ranking for depression level among different IBS subtypes and healthy controls

**Fig. 8:**
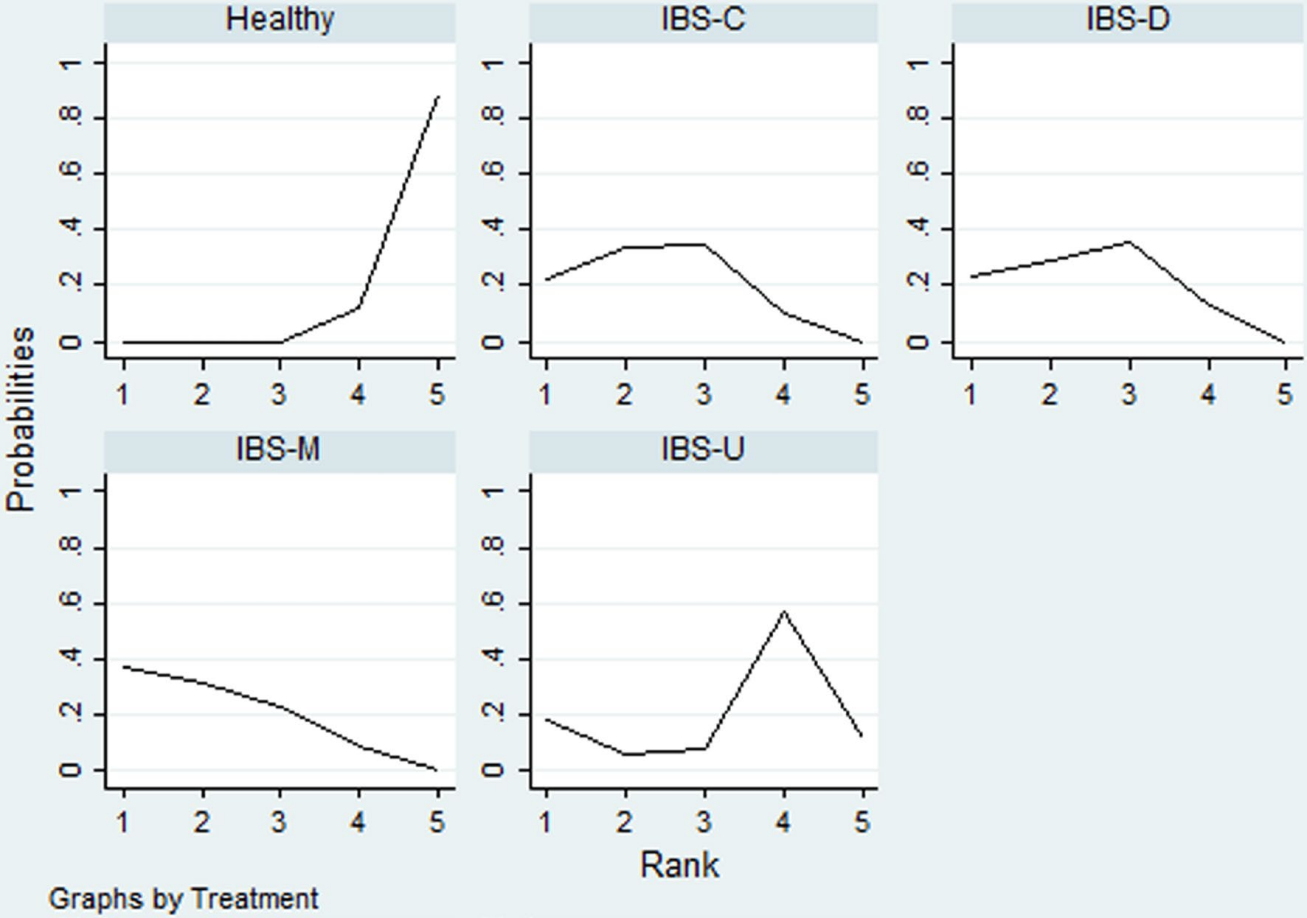
Ranking for anxiety level among different IBS subtypes and healthy controls

Table 4 showed, for each IBS subtypes, the likelihood with the highest levels of depression and anxiety. Rank probability indicated that IBS-M had the highest levels of depression, followed by IBS-C, IBS-D, IBS-U, healthy controls (Fig. 7), and the levels of anxiety in different IBS subtypes and healthy controls showed the same probability with depression level (Fig. 8).

**Table 4:** Rank probability of SUCRA

Treatment	Depression		Anxiety	
	SUCRA	Mean rank	SUCRA	Mean rank
Healthy	6.1	4.8	3	4.9
IBS-C	75.9	2	67.1	2.3
IBS-D	61.7	2.5	65.5	2.4
IBS-M	78.2	1.9	74.2	2
IBS-U	28	3.9	40.3	3.4

### The prevalence of depression and anxiety in IBS patients

**Fig. 9:**
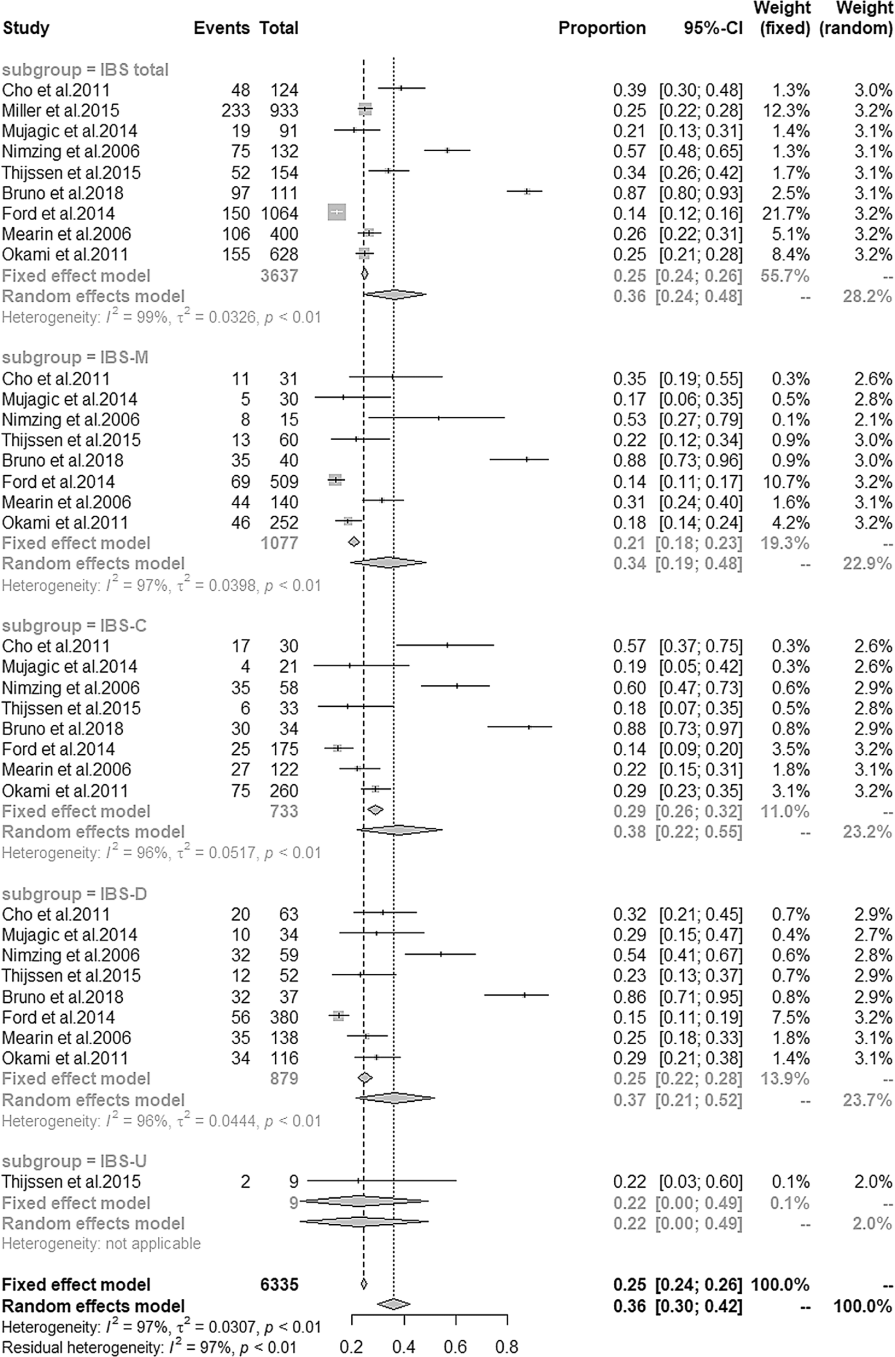
Prevalence of depression among different IBS subtypes

**Fig. 10:**
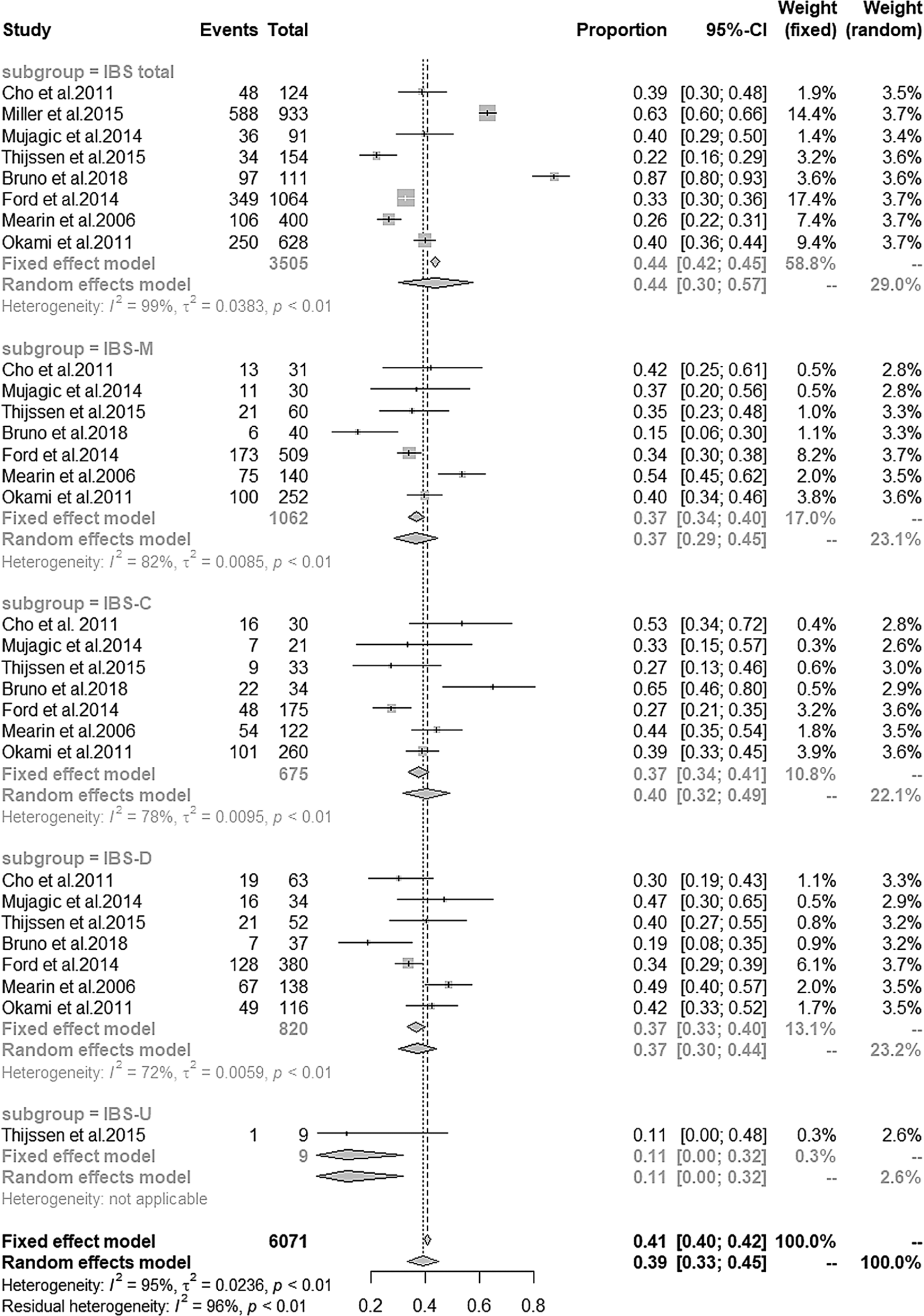
Prevalence of anxiety among different IBS subtypes

Nine studies [18, 21, 27, 29, 30–32, 34, 35] reported the prevalence of depression and anxiety in IBS patients. The details can be found in Table 5. Single-arm meta analysis showed the prevalence of depression and anxiety in total IBS was 36% and 44% respectively. Subgroup analyses showed the prevalence of depression in IBS-M, IBS-C, IBS-D, IBS-U was 34%, 38%, 37% and 22% respectively (Fig. 9), and anxiety prevalence in IBS-M, IBS-C, IBS-D, IBS-U was 37%, 40%, 37% and 11% respectively (Fig. 10).

**Table 5:** The prevalence of depression and anxiety in IBS patients

Study	Depression (%)						Anxiety (%)					
	Diagnostic criteria	IBS total	IBS-M	IBS-C	IBS-D	IBS-U	Diagnostic criteria	IBS total	IBS-M	IBS-C	IBS-D	IBS-U
Cho et al. [27]	HADS score ≥ 8	38.6	35.5	56.7	31.7	NA	HADS score ≥ 8	38.6	41.9	53.3	30.2	NA
Miller et al. [21]	HADS score ≥ 10	25	NA	NA	NA	NA	HADS score ≥ 10	63	NA	NA	NA	NA
Mujagic et al. [34]	HADS score ≥ 8	20.5	17.1	20	28.6	NA	HADS score ≥ 8	39.8	35.7	35	45.7	NA
Ladep et al. [35]	DSM-IV	56.8	53.3	61	53.4	NA	NA	NA	NA	NA	NA	NA
Thijssen et al. [18]	HADS score ≥ 8	NA	22	17	23	NA	HADS score ≥ 8	NA	35	27	4	11
Bruno et al. [30]	HARD ≥ 8	87.4	87.5	88.2	86.5	NA	HRSA ≥ 17	87.4	15	64.7	18.9	NA
Ford et al. [29]	HADS ≥ 11	14.1	13.6	14.3	14.7	NA	HADS ≥ 11	32.8	34	27.4	33.7	NA
Mearin et al. [31]	EuroQoL-5D	26.5	31.4	22.5	25.3	NA	EuroQoL-5D	26.5	53.7	44.4	48.9	NA
Okami et al. [32]	HADS ≥ 11	24.7	18.3	28.8	29.3	NA	HADS ≥ 11	39.8	39.7	38.8	42.2	NA

NA, not applicable; HADS, Hospital Anxiety and Depression Scale; MMPI, scales of the Minnesota Multiphasic Personality Inventory; DSM, diagnostic and statistical manual of mental disorders; HRSD, Hamilton Rating Scale for depression; HRSA, Hamilton Rating Scale for anxiety

## Discussion

To our knowledge, this study was the first network meta-analysis that explored the level and prevalence of depression or anxiety among healthy controls and different IBS subtypes patients. In this network meta analysis, 18 studies with a total of 7095 participants were included. Healthy controls had a lower level depression and anxiety than all IBS subtypes, but no significant difference was found between IBS-U and healthy controls in depression level. No significant difference was found in the level of depression and anxiety among different IBS subtypes. Ranking probability showed that IBS-M was associated with the highest level of depression and anxiety symptoms, followed by IBS-C/IBS-D and IBS-U. Single-arm meta-analysis showed that the prevalence of depression and anxiety was highest in IBS-C (38 and 40% respectively) and lowest in IBS-U (22 and 11% respectively).

Psychological factors play an important role in the development and maintenance of IBS, and have high correlation with IBS patients’ symptom severity, treatment response, sick cycle. It has been investigated by previous studies that IBS patients were more likely to suffer from depression and anxiety than healthy controls [7, 11–13]. But these studies did not distinguish the differences explicitly among different IBS subtypes in the comorbidities or level of depression and anxiety. This study investigated which subtypes of IBS were likely to be comorbid with depression and anxiety and provide useful information for health providers to make appropriate decisions. Based on direct and indirect comparing, no significant difference was found between IBS-U and healthy controls in depression level. The result was in conflict with the indirect comparison. We found that only one study [25] reported the data and the direct comparison pooled effect size was only − 0.7, indicating that even if there was a statistical difference, the difference was small and may be caused by statistical deviation. Therefore, it can be judged that there was no difference in anxiety level between healthy controls and IBS-U. We suggest that adequate psychological screening and appropriate psychotherapy are demanded for patients of IBS-C, IBS-D and IBS-M.

Another important finding of this study was the confirmation of no significant difference in the level of depression and anxiety among different IBS subtypes. But the ranking probability showed that the IBS-M was associated with the highest level of depression and anxiety. The result was inconsistent with the previous pairwise meta-analysis published in 2017 [10]. The meta-analysis indicated that the pooled SMDs of depression and anxiety levels were highest in IBS-C (0.83 and 0.81). We found that the samples used in the subgroup analysis in that meta-analysis were smaller than our study, it seemed that our results were more reliable. With respect to the prevalence of depression and anxiety in IBS patients, our single-arm meta-analysis showed that the prevalence of both depression and anxiety in total IBS patients was more than 20%, which was consistent with the study published in 2019[13]. What’s more, IBS-U was firstly included in this meta-analysis, the prevalence of anxiety among different IBS subtypes was also conducted.

There were some limitations in our systematic review. First, different study types were involved including cohort, case control and cross-sectional study. Second there were different versions of IBS diagnose criteria and mental disorders criteria. Third, different measurement scales were used in the included studies including HRSD, HRSA, MPPI, BDI, and HADS. In this study, 8 scores of HADS were defined as the cut-off point of mental disorders in three studies [18, 27, 34], 10 scores of HADS as the cut-off point in one study [21], 11 scores of HADS as the cut-off point in two study [29, 32]. It was considered that the cut-off of eight scores on the HADS showed the most optimal benefit between sensitivity and specificity [29]. Besides, the chronic illness is also a major risk factor for depression and increases the duration of depressive episode [31]. Symptom-screening questionnaire such as HADS may cause bias for overestimating or underestimating the psychological severity of medically ill patients [36], which leading to the misdiagnosis or over diagnosis of psychological disorders. So we suggest using different psychological scales and proper cut-off point to reflect the patients’ mental conditions.

Through this study, big heterogeneities were found among existed studies focused on the subtypes of IBS and mental disorders. We suggested high-quality community-based cross-sectional study surveying the level of depression and anxiety in patients with different subtypes of IBS should be carried out. Depression and anxiety assessments should be provided by clinical service, as there were a high prevalence and low diagnosis and treatment rate of depressive and anxiety symptoms in patients with IBS [37]. As depression or anxiety can show noteworthy phases changing even under effective treatments [38], we also recommend continuously monitoring the patient's psychological level to take control of the progress of the disease treatment.

## Conclusion

Based on the available evidence, healthy controls had a lower level of depression and anxiety than all IBS subtypes, but no significant difference was found between IBS-U and healthy control in depression level. No significant difference was found in the level of depression and anxiety among different IBS subtypes. Ranking probability results showed that IBS-M was associated with higher depression and anxiety level. Adequate psychological screening and appropriate psychotherapy are more suitable and needed for patients of IBS-C, IBS-D and IBS-M instead of IBS-U. Further studies on this topic should be carried out.

## Data Availability

The data that support the findings of this study are available from the corresponding author upon reasonable request.

## Appendix 1: Search strategy

### PubMed via Ovid

#1 Irritable Bowel Syndrome [Mesh].

#2 irritable bowel syndrome [Title/Abstract].

#3 #1 OR #2.

#4 depression [Mesh].

#5 depressive disorder [Mesh].

#6 anxiety [Mesh].

#7 anxiety disorders[Mesh].

#8 depression[Title/Abstract].

#9 depressive disorder*[Title/Abstract].

#10 dysthymic disorder*[Title/Abstract].

#11 melancholia[Title/Abstract].

#12 despondency[Title/Abstract].

#13 dysthymic disorder*[Title/Abstract].

#14 dysthymia[Title/Abstract].

#15 melancholy[Title/Abstract].

#16 sadness[Title/Abstract].

#17 low mood[Title/Abstract].

#18 despair[Title/Abstract].

#19 anxiety[Title/Abstract].

#20 anxiety disorder*[Title/Abstract].

#21 anxiousness[Title/Abstract].

#22 angst[Title/Abstract].

#23 apprehension[Title/Abstract].

#24 fear[Title/Abstract].

#25 nervousness[Title/Abstract].

#26 dysphoria[Title/Abstract].

#27 mood disorder*[Title/Abstract].

#28 affective disorder*[Title/Abstract].

#29 #4 OR #5 OR #6 OR #7 OR #8 OR #9 OR #10 OR #11 OR #12 OR #13 OR #14 OR #15 OR #16 OR #17 OR #18 OR #19 OR #20 OR #21 OR #22 OR #23 OR #24 OR #25 OR #26 OR #27 OR #28.

#30 #3 AND #29.

### EMBASE via Ovid

#1 ‘Irritable Bowel Syndrome’/exp.

#2 ‘irritable bowel syndrome’: ti,ab,kw.

#3 #1 OR #2.

#4 ‘depression’ /exp.

#5 ‘depressive disorder’ /exp.

#6 ‘anxiety’ /exp.

#7 ‘anxiety disorders’ /exp.

#8 ‘depression’: ti,ab,kw.

#9 ‘depressive disorder*’: ti,ab,kw.

#10 ‘dysthymic disorder*’: ti,ab,kw.

#11 ‘melancholia’: ti,ab,kw.

#12 ‘despondency’: ti,ab,kw.

#13 ‘dysthymic disorder*’: ti,ab,kw.

#14 ‘dysthymia’: ti,ab,kw.

#15 ‘melancholy’: ti,ab,kw.

#16 ‘sadness’: ti,ab,kw.

#17 ‘low mood’: ti,ab,kw.

#18 ‘despair’: ti,ab,kw.

#19 ‘anxiety’: ti,ab,kw.

#20 ‘anxiety disorder*’: ti,ab,kw.

#21 ‘anxiousness’: ti,ab,kw.

#22 ‘angst’: ti,ab,kw.

#23 ‘apprehension’: ti,ab,kw.

#24 ‘fear’: ti,ab,kw.

#25 ‘nervousness’: ti,ab,kw.

#26 ‘dysphoria’: ti,ab,kw.

#27 ‘mood disorder*’: ti,ab,kw.

#28 ‘affective disorder*’: ti,ab,kw.

#29 #4 OR #5 OR #6 OR #7 OR #8 OR #9 OR #10 OR #11 OR #12 OR #13 OR #14 OR #15 OR #16 OR #17 OR #18 OR #19 OR #20 OR #21 OR #22 OR #23 OR #24 OR #25 OR #26. OR #27 OR #28.

#30 #3 AND #29.

### Cochrane library

#1 MeSH descriptor: [Irritable Bowel Syndrome].

#2 irritable bowel syndrome: ti,ab,kw.

#3 #1 OR #2.

#4 MeSH descriptor: [depression].

#5 MeSH descriptor: [depressive disorder].

#6 MeSH descriptor: [anxiety].

#7 MeSH descriptor: [anxiety disorders].

#8 depression: ti,ab,kw.

#9 depressive disorder*: ti,ab,kw.

#10 dysthymic disorder*: ti,ab,kw.

#11 melancholia: ti,ab,kw.

#12 despondency: ti,ab,kw.

#13 dysthymic disorder*: ti,ab,kw.

#14 dysthymia: ti,ab,kw.

#15 melancholy: ti,ab,kw.

#16 sadness: ti,ab,kw.

#17 low mood: ti,ab,kw.

#18 despair: ti,ab,kw.

#19 anxiety: ti,ab,kw.

#20 anxiety disorder*: ti,ab,kw.

#21 anxiousness: ti,ab,kw.

#22 angst: ti,ab,kw.

#23 apprehension: ti,ab,kw.

#24 fear: ti,ab,kw.

#25 nervousness: ti,ab,kw.

#26 dysphoria: ti,ab,kw.

#27 mood disorder*: ti,ab,kw.

#28 affective disorder*: ti,ab,kw.

#29 #4 OR #5 OR #6 OR #7 OR #8 OR #9 OR #10 OR #11 OR #12 OR #13 OR #14 OR #15 OR #16 OR #17 OR #18 OR #19 OR #20 OR #21 OR #22 OR #23 OR #24 OR #25 OR #26. OR #27 OR #28.

#30 #3 AND #29.

## Abbreviations

IBS: irritable bowel syndrome
SMD: standardized mean difference
IBS-D: IBS with diarrhea
IBS-C: IBS with constipation
IBS-M: IBS with mixed symptoms of constipation and diarrhea
IBS-U: unsubtyped IBS
FGIDs: functional gastrointestinal disorders
HADS: the hospital anxiety and depression scale
HRSD: the Hamilton rating scale for depression
HRSA: the Hamilton rating scale for anxiety
BDI: the Beck's depression inventory
SDS: the self-rating depression scale
NOS: the Newcastle–Ottawa scale
AHRQ: the agency for healthcare research and quality
SUCRA: the surface under the cumulative ranking curve
